# Students’ Histology : A Course of Normal Histology for Students and Practitioners of Medicine

**Published:** 1899-02

**Authors:** 


STUDENTS' HISTOLOGY : A Course of Normal Histology for Students and Practitioners of Medicine. By MAURICE U. MILLER, M. D., late Director of the Department of Normal Histology in Loomis's Laboratory, University of the City of New York. Revised by HERBERT U. WILLIAMS, M. D., Professor of Pathology and Bacteriology, Medical Department, University of Buffalo. Third revised edition. Profusely illustrated. New York : William Wood & Co. 1898.

The present tendencies in medicine require more and more the application of the microscope as an aid to diagnosis. Its intelligent use demands practical laboratory training rather than extended theoretical knowledge. Miller's histology aims to present the subject from the viewpoint of the beginner rather than that of the advanced student or teacher ; moreover, the work is intended to be used in the laboratory. Throughout the book are numerous "practical demonstrations" giving directions for the preparation of specimens, and the powers of the microscope to be used in their study and also calling attention to the salient features to be observed. This edition became necessary because of the advances of histology during the past ten years and also because of the increased demand for laboratory work. The text has been thoroughly revised and brought up to date, involving the addition of enough new material to increase the number of pages from 214 to 259. The illustrations have also been increased in number from 126 to 158, besides many of the old being replaced by new. All are clearer than those in the second edition. The book is also better printed on better paper and better bound than was the old. It is divided into three parts: I. Technology; II. Structural elements; III. Organs.

The first part consists of two sub-heads (1) The laboratory microscope; (2) Preparation of tissue for microscopical purposes.

The description of the microscope and directions for its use could be improved by more elaborate details. The preparation of tissue includes teasing, sectioning, fixing, hardening, embedding (both paraffin and celloidin) staining, mounting and ends with a paragraph on the care of the microscope. Some of the methods of section-cutting are now we believe little used, but the directions for hardening, embedding, staining and mounting are only those most approved and generally applicable. These are made still more useful by frequent reference throughout the book. The directions for staining are particularly clear.

Part second, structural elements, beginning with the preliminary study of extraneous substance and the like, takes up the cell epithelium, fibrous connective tissue, cartilage, bone, special connective tissue, muscular tissue and blood. This last, especially well-written and greatly enlarged, is very opportune just now, initiating the beginner in the methods of blood examination, so important in diagnosis.

Part third, organs, consists of sections on skin, circulatory system, lymphatic system, spleen, thymus, respiratory organs, teeth, glands, alimentary canal, liver, kidney, female and male generative organs, suprarenal body, nervous system. These have all been carefully revised and some rewritten. The arrangement has also been much improved. The section on the nervous system has been entirely rewritten, making it one of the clearest preliminary surveys of this topic. This revision of Miller makes it one of the best books for its purpose on the market. For the student beginning the study of histology in the laboratory, or by himself, it is indispensable. By its use he will be able to take up understandingly the advanced study of the subject, but more particularly will he be prepared for pathology. A. T. K., Jr.

